# Design and Synthesis of Dendrimers with Facile Surface Group Functionalization, and an Evaluation of Their Bactericidal Efficacy

**DOI:** 10.3390/molecules22060868

**Published:** 2017-05-24

**Authors:** Elizabeth Ladd, Amir Sheikhi, Na Li, Theo G.M. van de Ven, Ashok Kakkar

**Affiliations:** 1 Department of Chemistry, McGill University, 801 Sherbrooke St. West, Montreal, QC H3A 0B8, Canada; elizabeth.ladd@mail.mcgill.ca (E.L.); amir.sheikhi@mail.mcgill.ca (A.S.); Blinan04st@gmail.com (N.L.); 2 Pulp and Paper Research Centre, McGill University, 3420 rue University, Montreal, QC H3A 2A7, Canada; 3 Centre for Self-Assembled Chemical Structures, McGill University, 801 Sherbrooke St. West, Montreal, QC H3A 0B8, Canada

**Keywords:** dendrimers, hyperbranched macromolecules, synthesis, click chemistry, bactericide

## Abstract

We report a versatile divergent methodology to construct dendrimers from a tetrafunctional core, utilizing the robust copper(I) catalyzed alkyne-azide cycloaddition (CuAAC, “click”) reaction for both dendrimer synthesis and post-synthesis functionalization. Dendrimers of generations 1–3 with 8–32 protected or free OH and acetylene surface groups, were synthesized using building blocks that included acetylene- or azide-terminated molecules with carboxylic acid or diol end groups, respectively. The acetylene surface groups were subsequently used to covalently link cationic amino groups. A preliminary evaluation indicated that the generation one dendrimer with terminal NH_3_^+^ groups was the most effective bactericide, and it was more potent than several previously studied dendrimers. Our results suggest that size, functional end groups and hydrophilicity are important parameters to consider in designing efficient antimicrobial dendrimers.

## 1. Introduction

Dendrimers are well-defined hyperbranched macromolecules in which structural regularity arises from the controlled layer-by-layer build-up, with each additional layer leading to an exponential increase in the number of surface groups [1,2,3,4,5,6,7]. The overall structure and properties of dendrimers get contributions from their core, backbone, and the surface groups at the periphery [8]. The number of reactive ends at the core molecule influences the density of end units at each generation that primarily interact with the outside environment [9]. For example, a third generation dendrimer with a tetrafunctional core has 32 surface groups, while a corresponding dendrimer with a trifunctional core has 24. Flexibility of both the core and backbone also has a significant effect on the conformation of the structure [10,11]. A rigid backbone of the dendrimer leads to an open structure, while more flexible core and backbones correspond to more compact, globular dendrimers, as the arms can fold back on themselves [12,13]. The synthetic versatility and our ability to tailor their surfaces are some of the features, which make these monodisperse macromolecules attractive for a wide variety of applications.

One of the topical areas of interest in which dendrimers are becoming increasingly important is their ability to act as bactericides [14]. Multivalent surfaces of these hyperbranched macromolecules offer opportunities to tailor the efficacy of antimicrobial agents using a single scaffold [15,16,17,18,19]. Cationic amino groups are well known to exert bactericidal effect by disrupting the integrity of bacterial cell membranes, eventually leading to cell lysis and death [18,20]. It has been shown that amine- and ammonium-terminated carbosilane dendrimers act as bactericides against Gram-positive and -negative bacteria, with almost two orders of magnitude less minimum inhibitory concentration (MIC) and minimum bactericidal concentration (MBC), than their monofunctional counterparts. However, these dendrimers can only be solubilized in water when a small amount of DMSO (1%) is added [21]. In another study, polyamidoamine (PAMAM) dendrimers were functionalized with nitric oxide (NO) releasing quaternary ammonium groups, which showed enhanced bactericidal activity and anti-biofilm formation properties as compared to non-NO releasing counterparts [22,23]. Hyaluronic acid/PAMAM multilayered dendrimers have also been used as antibacterial materials [24]. These studies suggest that by carefully designing dendrimers, one could achieve high efficacy through tailoring of desired functions [25].

We have developed a versatile synthetic methodology to dendrimers utilizing highly efficient alkyne-azide “click” chemistry [26,27,28], which leads to surface terminated OH or acetylene groups. The synthesis of dendrimers required the design of multifunctional building blocks including the one bearing a diol at one end and an azide at the other. The azide can be clicked to the peripheral alkynes of the growing dendrimer, regenerating the original alcohol functionality. The sequence can then be repeated to construct subsequent generations in a divergent manner. These dendrimers provide a facile method to introduce a variety of desired peripheral moieties, and we demonstrate the simplicity of this approach by functionalizing the surfaces of these dendrimers with cationic amino groups. We have evaluated the potential of these water-soluble cationic dendrimers for bactericidal activity, and we demonstrate that a balance of size, the terminal end groups and hydrophilicity plays an important role in their efficacy. Dendrimers functionalized with cationic amino groups were found to be highly potent antimicrobial agents, and the generation 1 dendrimer with surface NH_3_^+^ groups was the most efficient bactericide among such macromolecules.

## 2. Results and Discussion

### 2.1. Synthesis

**Scheme 1:**
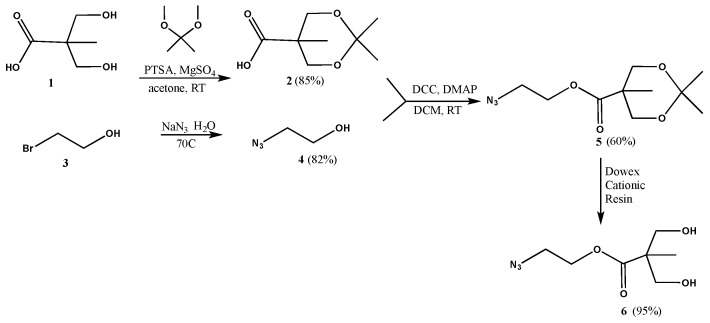
Synthesis of building blocks.

The synthesis of dendrimers was carried out using a careful design of building blocks with compatible functionalities. Since we intended to employ click chemistry both in the synthesis and functionalization of our dendrimers, we selected two building units, one with an acetylene moiety and another with an azide. The azide functionalized 2,2-bis(hydroxymethyl)propanoic acid (bis-MPA, Scheme 1) was prepared from bis-MPA (**1**) and bromoethanol (**3**). The diol terminal of the bis-MPA unit was first protected with an acetonide group, to yield **2**, which could be purified easily by neutralizing with *p*-toluenesulfonic acid, followed by filtration.

**Scheme 2:**
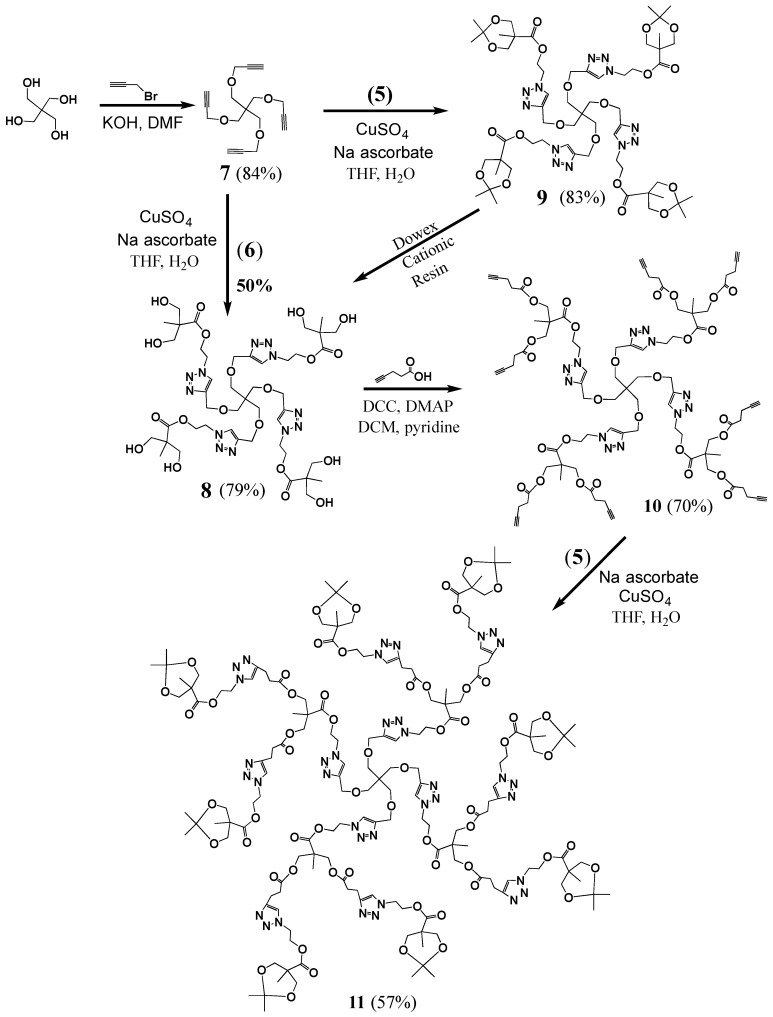
Synthesis of G0–G2 dendrimers.

Bromoethanol (**3**) was reacted with sodium azide to yield **4**, which was then coupled with **2** using *N*,*N*′-dicyclohexylcarbodiimide (DCC)/4-dimethylaminopyridine (DMAP), to yield **5**, a bifunctional molecule with a protected diol at one end and an azide at the other. It was deprotected to yield **6**, with azide and diol terminals. Propargyl bromide was coupled to the pentaerythritol core, yielding the tetra-acetylene terminated G0 dendrimer **7** (Scheme 2). The azide functionalized bis-MPA **6** was then clicked to G0 using CuAAC reaction to yield G1 dendrimer **8** with eight terminal hydroxide groups. It can also be prepared by first reacting core **7** with protected version of the azide functionalized bis-MPA **5** using CuAAC reaction to give **9**, followed by deprotection. This method gave better yields than the first route.

**Scheme 3:**
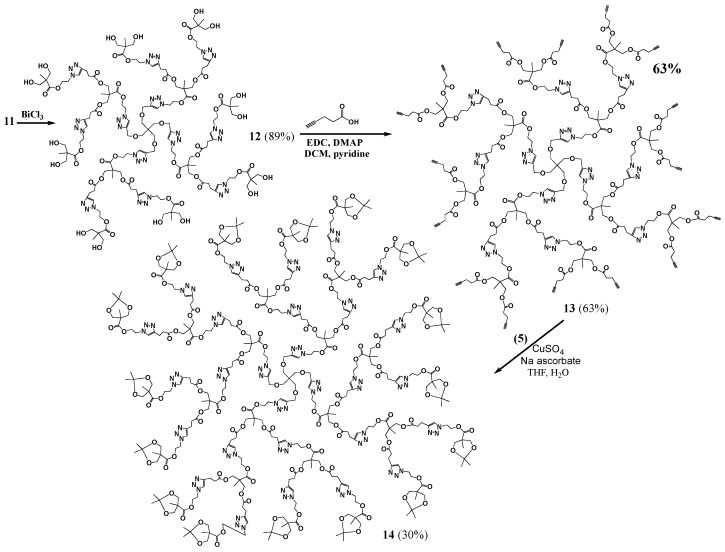
Synthesis of G2-G3 dendrimers.

**Scheme 4:**
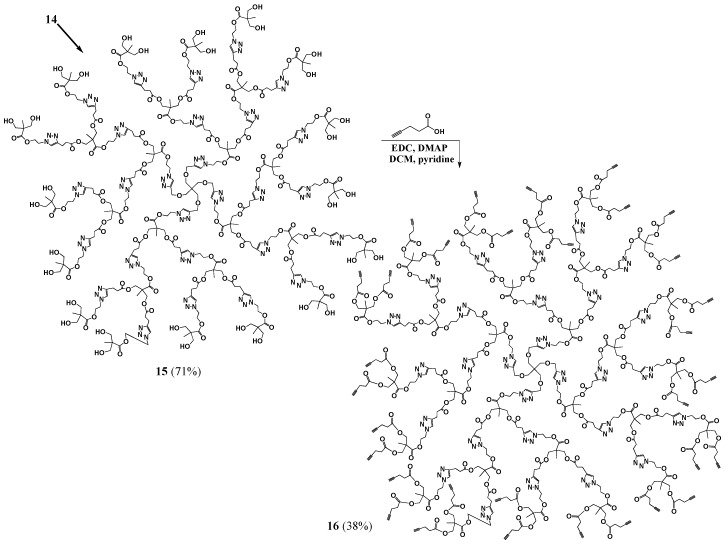
Synthesis of G3 dendrimer with terminal OH and acetylene groups.

Several attempts to covalently link propargyl bromide to G1 dendrimer **8** using a similar procedure to that used to prepare G0 **7** failed. Since the important component was that the structure has acetylene termini, we considered a variation on this structure. An esterification with 4-pentynoic acid was carried out to prepare G1 (compound **10** with eight terminal acetylene groups). Compound **5** was then clicked onto the dendrimer to yield protected G2 dendrimer (p-G2, **11**), with sixteen terminal protected hydroxides, which was purified by dialysis. The deprotection using bismuth trichloride was employed to give the desired hydroxide terminated G2 dendrimer. A simple filtration to remove the remaining salts was the only purification necessary to yield G2 (**12**, Scheme 3). Another esterification with 4-pentynoic acid was then carried out to generate **13**, with sixteen terminal acetylenes. The purification of **13** was quite simple, employing only washing and filtration steps. A click reaction was then used to attach an additional layer of **5** onto **13**, generating pG3 **14**, with protected hydroxide groups, which was purified by precipitation. The compound was then deprotected to yield G3 (**15**, Scheme 4) with thirty two terminal hydroxyls, which were used to introduce terminal acetylene groups through esterification (**16**, Scheme 4).

**Scheme 5:**
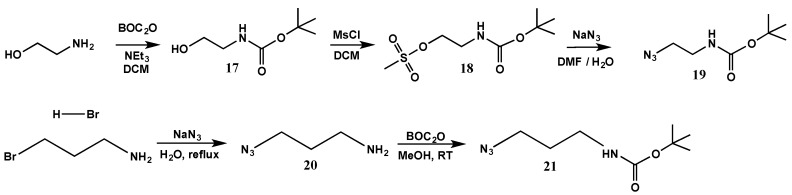
Synthesis of protected amine-azides.

Dendrimers with positively charged amine groups on the surface were synthesized from the acetylene terminated dendrimers, by reacting them with protected amino-azides. The latter were synthesized from the commercially available amino alcohol. The amine was first protected with a t-butoxycarbonyl (BOC) group to yield **17** (Scheme 5), which was purified by washing in sequence with a basic followed by an acidic solution, brine and finally water. The alcohol group of **17** was then converted to a mesylate upon reaction with methane sulfonyl chloride to yield **18**, which was subsequently azidified to afford **19**. The amino-azide with a longer arm was prepared from commercially available amino-bromide by reacting it with sodium azide to yield **20**, which was purified by a basic work-up, followed by extraction (Scheme 5). The amine moiety of **20** was then protected with ^t^BOC to yield **21**.

**Figure 1:**
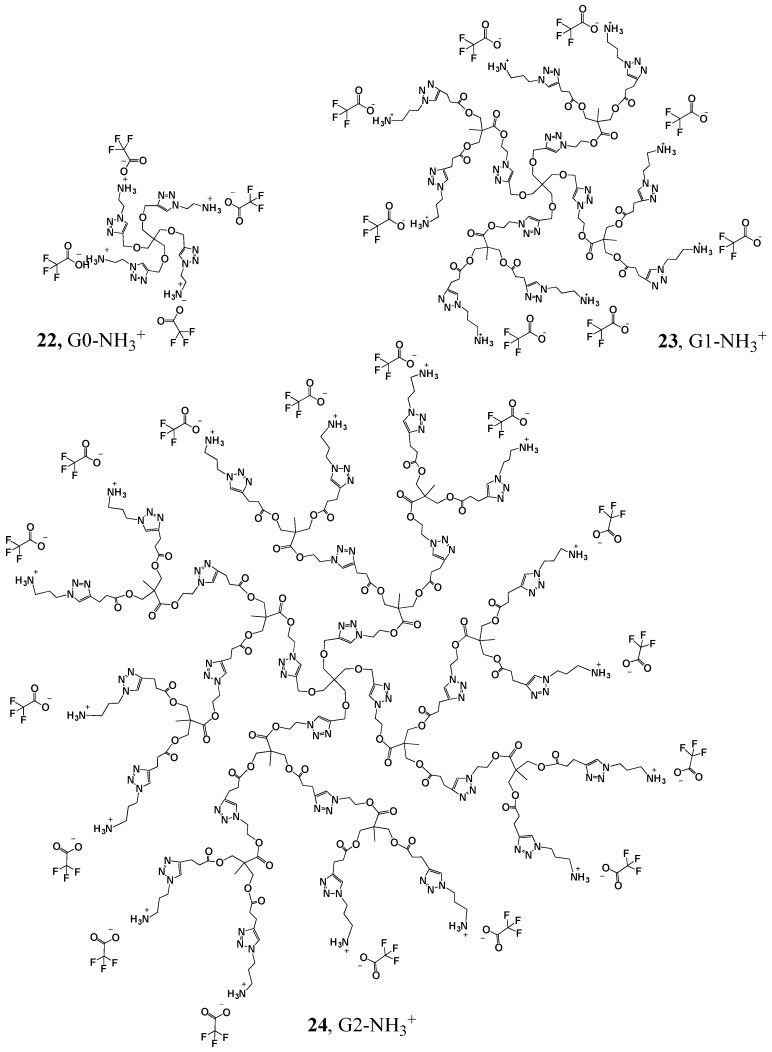
Dendrimers with positively charged surface groups.

The protected amine-azides **19** and **21** were clicked on to the acetylene terminated dendrimers **7**, **10** and **13**, and the surface amine groups on the resulting dendrimers were deprotected using trifluoroacetic acid (TFA), to yield the desired cationic amino terminated dendrimers (Figure 1: G0-NH_3_^+^ **22**, G1-NH_3_^+^ **23**, and G2-NH_3_^+^ **24**). We used amine **21** that was one carbon longer for G1-2 dendrimers to reduce any steric hinderance on the surface for the larger dendrimers.

### 2.2. Bactericidal Activity of Dendrimers

**Figure 2:**
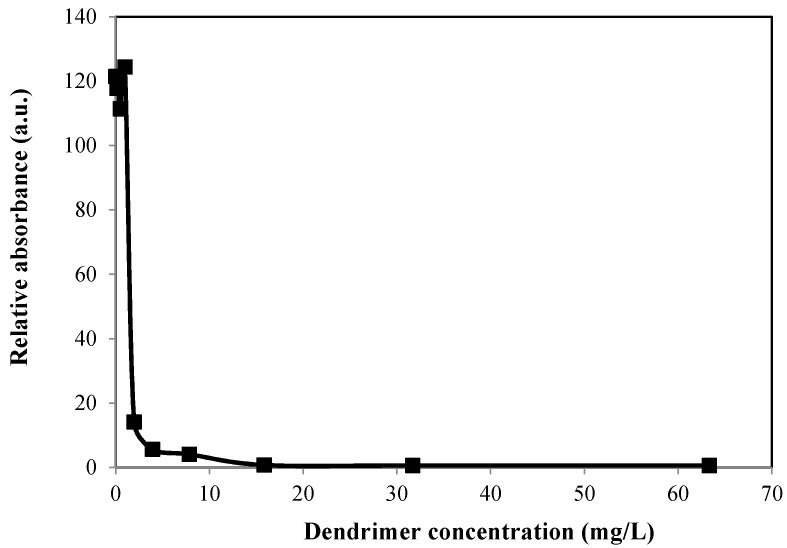
Relative absorbance versus dendrimer concentration curve for the determination of MIC for G2-NH_3_^+^.

We explored the bactericidal efficacy of the cationic amine terminated dendrimers, and one of the hydroxide terminated water soluble dendrimer ((G1-OH), Figure 1) for comparision purposes, as it is known that hydroxide terminated molecules may also show bactericidal effect [20]. Bactericidal efficacy was assessed using two parameters: minimum inhibitory concentration (MIC), and minimum bactericidal concentration (MBC). MIC is the lowest concentration of the antimicrobial (in this study, the dendrimer) that inhibits the growth of a microorganism after overnight incubation. It gives a measure of the inhibition of the growth of bacteria as a function of concentration. MBC is the lowest concentration of the dendrimer that will prevent the growth of bacteria, and MBC is always larger than MIC. Aqueous solutions of dendrimers with a range of different concentrations were prepared, and then added to aliquots of bacteria solution. *Escherichia coli* (*E. coli*) with standard Gram-negative ATCC 11229^TM^ strain were chosen for bactericidal activity experiments in this study, as these are the most widely studied microorganisms, and have a much faster growth rate which simplifies the time scale of the experiments. The initial optical density (OD) of the solutions was measured, and they were subsequently incubated overnight. After 18–24 h had elapsed, the OD was measured again. The relative absorbance of solutions as a function of bactericide concentration for G2-NH_3_^+^ is shown in Figure 2. The minimum inhibitory concentration (MIC) was determined as the lowest concentration point where the curve drops sharply to zero.

The bactericidal efficacies of four different dendrimers, three of them functionalized with cationic amino groups (G0-NH_3_^+^, G1-NH_3_^+^ & G2-NH_3_^+^), and the fourth with hydroxide surface groups (G1-OH) are shown in Table 1. The most efficient bactericide was the generation one cationic amine terminated dendrimer, G1-NH_3_^+^ (MIC = 0.9 mg/L, 8 NH_3_^+^ groups, Table 1), and the least effective was the smallest structure, G0-NH_3_^+^ (MIC = 32–64 mg/L, 4 NH_3_^+^ groups, Table 1). Dendrimer G1-OH was found to have no bactericidal action at any of the concentrations tested (MIC > 64 mg/L, 8 OH groups, Table 1), which clearly suggests the importance of cationic surface groups for bactericidal activity. Since G1-NH_3_^+^ was more effective in its bactericidal activity than both the lower (G0-NH_3_^+^ 4 NH_3_^+^ groups, MIC = 32–64 mg/L,) and higher (G2-NH_3_^+^ 16 NH_3_^+^ groups, MIC = 1–16 mg/L) generation dendrimers with the same backbone and type of end groups, it indicated that G1-NH_3_^+^ provides the best balance between the number of bactericidal end groups and biopermeability. These results may also suggest that the generation 2 dendrimer is past the tipping point where the gain in bactericidal activity obtained from additional charged end groups, is outweighed by the loss of biopermeability. The MBC for G1-NH_3_^+^ (4–8 mg/L, 8 NH_3_^+^ groups, Table 1) followed the expected pattern of MBC > MIC for a given structure.

To better evaluate the bactericidal efficacy of our dendrimers, a comparison with dendrimers for which bactericidal activity has been reported earlier was carried out, and the data is presented in Table 2. The data corresponds only to dendrimers that function by the membrane disruptive mechanism (as opposed to anti-adhesive), where an MIC was measured [14]. Our best performing dendrimer, G1-NH_3_^+^ (8 NH_3_^+^ groups), is a more effective bactericide than any of the dendrimers (4–32 end groups) presented in Table 2. Comparable both in size and number of charged end groups to the generation 2 carbosilane dendrimer (MIC = 64 mg/L, 8 NMe_3_^+^ groups, Table 2) [21], G1-NH_3_^+^ (MIC = 0.9 mg/L, 8 NH_3_^+^ groups, Table 1) is more effective by almost two orders of magnitude. Equally comparable in size and number of charged end groups are the generation one carbosilane dendrimers (MIC = 4 mg/L, 4 NR_3_^+^ groups), and G0-NH_3_^+^ (MIC = 32–64 mg/L, 4 NR_3_^+^ groups, Table 1). Interestingly, however, the trend is reversed in this case, with the generation one carbosilane dendrimer showed about an order of magnitude more bactericidal effect than G0-NH_3_^+^. Since both sets of structures present the same number of charged amino groups, approximately the same molecular weight, and were tested against the same bacteria, the only major difference between the two dendrimer backbones seems to be in their hydrophilicity. It is known that structures which present more hydrophobic units can permeate the bacterial structure more easily to arrive at their site of action [14]. These results may suggest that for dendrimers with hydrophilic backbones, such as the ones presented here, the peak bactericidal efficiency is reached at a higher generation than for dendrimers with hydrophobic backbones, such as carbosilane.

The bactericidal efficacy of the neutral amine terminated generation three PAMAM dendrimer (MIC = 6.3 mg/L, 31 NH_2_ groups Table 2) and generation five (12.5 mg/L, 110 NH_2_ groups), both fall into the wide range determined for our G2-NH_3_^+^ (MIC = 1–16 mg/L, 16 NH_3_^+^ groups, Table 1) dendrimer. The molecular mass of G2-NH_3_^+^ is comparable to that of the generation three PAMAM dendrimer. It is difficult to draw definite conclusions about these results due to the fact that the structures have different backbones, number of end groups, type of end groups, and were tested against different bacteria. Interestingly, the neutral structures that were previously tested (generations three and five PAMAM dendrimers) both performed better than the only neutral structure tested in this study, G1-OH (MIC > 64 mg/L, 8 OH groups, Table 1).

Our results, as well as those for the dendrimers previously evaluated [29,30], indicate that higher generation number may not be a necessary and important factor for bactericidal action. Although a detailed evaluation is necessary in order to determine structure-property relationships, it should be noted that the largest structure, the generation 5 PAMAM dendrimer with 110 peripheral amino groups, is the least effective bactericide. This suggests that a balance between the size of the dendrimer and the number of end groups is a key factor.

## 3. Materials and Methods

### 3.1. Generaal Information

The reagents and solvents were purchased from Sigma-Aldrich (St. Louis, MO, USA) and Fisher Scientific (Hampton, NH, USA), and used as received. NMR spectral acquisitions were carried out on Mercury instruments (Varian, Palo Alto, CA, USA) and operated using VNMRJ 2.2D (Chempack 5) and VNMRJ 2.3A (Chempack 5) software using a 5 mm Smart Probe. The chemical shifts in ppm are reported relative to tetramethylsilane (TMS) as an internal standard for ^1^H, and ^13^C. Mass spectra analyses (HRMS, ESI) were performed and analysed on an Exactive Plus Orbitrap-API (Thermo Scientific, Waltham, MA, USA) high resolution mass spectrometer and on MALDI Autoflex III-TOF (Bruker, Billerica, MA, USA). Procedures for the MIC and MBC determination were followed as described in [31]. The Compounds 2,2,5-trimethyl-1,3-dioxane-5-carboxylic acid (**2**) [32], azidoethanol [33], and propargylated pentaerythritol [34], were prepared by adopting and modifying the procedures reported earlier.

### 3.2. Building Blocks

*2,2,5-Trimethyl-1,3-dioxane-5-carboxylic acid* (**2**): *p*-Toluenesulfonic acid (0.5975 g, 0.003141 mol) was added to a stirred solution of 2,2-bis(hydroxymethyl)propanoic acid (**1**, 8.4265 g, 0.06282 mol) in acetone (34 mL) in a 250 mL round bottom flask, under nitrogen. 2,2-Dimethoxypropane (9.8141 g, 0.09423 mol) and magnesium sulfate (0.7562 g, 0.006282 mol) were then added, and the reaction mixture was left to stir for 48 h. A solution of ammonia in dioxane (6.27 mL, 0.5 M) was added to neutralize the acid. The crude mixture was filtered, and the solvent evaporated to yield the product as a white powder (9.30 g, 0.0534 mol, 85% yield). ^1^H-NMR (400 MHz, CDCl_3_): δ = 1.20 (s, 3H, -CO-C-CH_3_), 1.40 (s, 3H, -O-C-CH_3_), 1.43 (s, 3H, -O-C-CH_3_), 3.67 (t, 2H, -O-CH_2_-C-CO-), 4.18 (d, 2H, -O-CH_2_-C-CO-) ppm. ^13^C{^1^H}-NMR (300 MHz, CDCl_3_): δ = 18.42 (-CO-C-CH_3_), 22.03 (-O-C-CH_3_), 25.07 (-O-C-CH_3_), 41.71 (-CO-C-), 65.81 (-O-CH_2_-C-), 98.28 (-O-C-(CH_3_)_2_), 180.15 (-CO-) ppm.

Azidoethanol (**4**): A mixture of bromoethanol (**3**, 2.3742 g, 0.01900 mol) and sodium azide (2.4700 g, 0.03800 mol) in water (4 mL) was left stirring overnight at 70 °C. The reaction mixture was then extracted with DCM, the organic layer was isolated and dried with magnesium sulfate. The solvent was then evaporated to yield the product as yellow oil (1.36 g, 0.01561 mol, 82% yield). ^1^H-NMR (400 MHz, CDCl_3_): δ = 3.46 (t, 2H, N_3_-CH_2_-CH_2_-), 3.79 (q, 2H, N_3_-CH_2_-CH_2_-) ppm. ^13^C{^1^H}-NMR (300 MHz, CDCl_3_): δ = 53.6 (N_3_-CH_2_-CH_2_-), 61.6 (N_3_-CH_2_-CH_2_-) ppm.

*2-Azidoethyl 2,2,5-trimethyl-1,3-dioxane-5-carboxylate* (**5**): A solution of azidoethanol (**4**, 0.74 g, 0.008498 mol), 2,2,5-trimethyl-1,3-dioxane-5-carboxylic acid (**2**, 2.2204 g, 0.01275 mol) and DMAP (0.5191 g, 0.004249 mol) in anhydrous DCM (10 mL) was stirred for 5 min under nitrogen. DCC (2.1041 g, 0.01020 mol) was then added to the reaction mixture, and stirred under nitrogen, at room temperature, overnight. The precipitate was filtered off, and the solvent was evaporated to yield a residue that was purified by column chromatography (1:7 ethyl acetate–hexanes) to yield the product as a white solid (1.25 g, 0.005139 mol, 60% yield). ^1^H-NMR (400 MHz, CDCl_3_): δ = 1.21 (s, 3H, -CO-C-CH_3_), 1.39 (s, 3H, -O-C-CH_3_), 1.44 (s, 3H, -O-C-CH_3_), 3.49 (t, 2H, N_3_-CH_2_-CH_2_-), 3.68 (d, 2H, -O-CH_2_-C-CO-), 4.21 (d, 2H, -O-CH_2_-C-CO-), 4.33 (t, 2H, N_3_-CH_2_-CH_2_-) ppm. ^13^C{^1^H}-NMR (300 MHz, CDCl_3_): δ = 18.5 (-CO-C-CH_3_), 22.3 (-O-C-CH_3_), 24.9 (-O-C-CH_3_), 42.0 (-C-CH_3_), 49.8 (N_3_-CH_2_-CH_2_-), 63.6 (N_3_-CH_2_-CH_2_-), 65.9 (-O-CH_2_-C-), 98.1 (-C-CH_3_), 174.0 (-CO-) ppm.

*2-Azidoethyl 3-hydroxy-2-(hydroxymethyl)-2-methylpropanoate* (**6**): A spoonful of Dowex cationic resin was added to a solution of **5** (0.301 g, 0.001728 mol) in methanol (6 mL). The mixture was left stirring overnight at room temperature. The supernatant was decanted off and the resin was rinsed several times with methanol. The supernatant was then combined with rinsing and the solvent was evaporated to yield the product as colorless oil (0.238 g, 0.001171 mol, 95% yield). ^1^H-NMR (400 MHz, CDCl_3_): δ = 1.09 (s, 3H, -CO-C-CH_3_), 2.47 (s, 2H, -OH), 3.41 (m, 2H, N_3_-CH_2_-CH_2_-), 3.50 (dd, 2H, -C-CH_2_-OH), 3.80 (dd, 2H, -C-CH_2_-OH), 4.28 (t, 2H, N_3_-CH_2_-CH_2_-) ppm. ^13^C{^1^H}-NMR (300 MHz, CDCl_3_): δ = 17.0 (CH_3_-C-), 49.3 (CH_3_-C), 49.8 (N_3_-CH_2_-CH_2_-), 63.5 (N_3_-CH_2_-CH_2_-), 68.2 (CH_2_-OH), 175.5 (-CO-) ppm.

Generation 0 (**7**): Anhydrous DMF (25 mL) was added by syringe to a round bottom flask containing pentaerythritol (2 g, 0.01469 mol) and KOH (12.5 g, 0.2228 mol), with stirring, under nitrogen. The mixture was stirred for 5 min, under nitrogen, at 0 °C. A solution of propargyl bromide (20 g, 0.1681 mol) in toluene (80%) was then added dropwise to the reaction mixture over 30 min. The reaction was heated at 40 °C overnight, under nitrogen. Water (100 mL) was added to the mixture, which was then extracted with diethyl ether (3 × 50 mL). The organic layers were isolated, combined, and washed with water (3 × 50 mL) and brine (3 × 50 mL). The organic layer was isolated and dried with sodium sulfate. The solvent was removed to yield an orange oil which was purified by column chromatography (2:8 ethyl acetate–hexanes) to give the product as an orange-brown solid (1.7468 g, 0.006058 mol, 84% yield). ^1^H-NMR (400 MHz, CDCl_3_): δ = 2.40 (t, 4H, CH-C-CH_2_-), 3.53 (s, 8H, -C-CH_2_-O-), 4.12 (d, 8H, CH-C-CH_2_-) ppm. ^13^C{^1^H}-NMR (300 MHz, CDCl_3_): δ = 44.7 (-C-CH_2_-O-), 58.7 (CH-C-CH_2_), 69.0 (-C-CH_2_-O-), 74.0 (CH-C-CH_2_), 80.0 (CH-C-CH_2_) ppm. ESI-MS: *m*/*z* = 311.1 [M + Na^+^].

Protected Generation 1 (**pG1**, **9**): A solution of CuSO_4_·5H_2_O (0.0191 g, 0.00007669 mol) in water (0.5 mL) was added to a round bottom flask containing a stirred solution of **7** (0.0768 g, 0.0002663 mol) and (**5**) (0.2683 g, 0.001103 mol) in tetrahydrofuran (THF) (0.5 mL). Sodium ascorbate (0.0317 g, 0.0001598 mol) was added, and the mixture was allowed to react overnight. The product was purified by column chromatography (100% methanol) to yield a yellow oil (0.2778 g, 0.0002202 mol, 83% yield). ^1^H-NMR (400 MHz, CD_3_OD): δ = 1.00 (s, 3H, -CO-C-CH_3_), 1.22 (s, 3H, -O-C-(CH_3_)_2_), 1.39 (s, 3H, -O-C-(CH_3_)_2_), 3.44 (s, 2H, -C-CH_2_-O-CH_2_), 3.63 (dd, 2H, -CO-C-CH_2_-O-), 4.07 (dd, 2H, -CO-C-CH_2_-O-), 4.51 (s, 2H, -O-CH_2_-C-N-), 4.54 (t, 2H, -N-CH_2_-CH_2_-O-), 4.72 (s, 2H, -N-CH_2_-CH_2_-O-), 8.04 (s, 1H, -C-CH-N-) ppm. ^13^C{^1^H}-NMR (300 MHz, CD_3_OD): δ = 17.19 (-C-CH_3_), 20.52 (-O-C-(CH_3_)_2_), 24.93 (-O-C-(CH_3_)_2_), 41.78 (-C-C-CH_3_), 45.11 (-C-CH_2_-O-), 48.90 (-N-CH_2_-CH_2_-O-), 62.63 (-N-CH_2_-CH_2_-O-), 64.06 (-CH_2_-O-CH_2_-C-N-), 65.47 (-C-CH_2_-O-C-), 68.65 (-C-CH_2_-O-CH_2_-C-N-), 97.97 (-O-C-(CH_3_)_2_), 124.07 (-C-CH-N-), 145.00 (-C-N-), 173.80 (-C-O-CH_2_-) ppm. MALDI-MS: *m*/*z* = 1267.3 [M + Li^+^].

Generation **1** (**G1**, **8**). A solution of CuSO_4_·5H_2_O (0.0191 g, 0.00007669 mol) in water (0.5 mL) was added to a round bottom flask containing a stirred solution of **7** (0.0768 g, 0.0002663 mol) and **6** (0.2597 g, 0.001278 mol) in THF (0.5 mL). Sodium ascorbate (0.0317 g, 0.0001598 mol) was then added and the mixture was allowed to react overnight. The product was purified by column chromatography (100% methanol) to yield an orange oil (0.1479 g, 0.0001343 mol, 50% yield).

**G1** (**8**) from **pG1** (**9**): Dowex Cationic Resin (6.72 g) was added to a solution of pG1 (**9**, 9.69 g, 0.0077 mol) in methanol (485 mL). The mixture was then left stirring overnight. The resin was then filtered off and the solvent evaporated to yield **8** as a viscous orange oil (6.68 g, 0.0061 mol, 79% yield). ^1^H-NMR (400 MHz, CD_3_OD): δ = 1.08 (s, 3H, -CO-C-CH_3_), 3.45 (s, 2H, -C-CH_2_-O-), 3.61 (q, 4H, -C-CH_2_-OH), 4.51 (t, 4H, -O-CH_2_-C-N-), 4.52 (s, 2H, -N-CH_2_-CH_2_-O-), 4.70 (t, 2H, -N-CH_2_-CH_2_-O-), 8.04 (s, 1H, -C-CH-N-) ppm. ^13^C{^1^H}-NMR (300 MHz, CD_3_OD): δ = 17.40 (-C-CH_3_), 46.53 (C-CH_2_-O-), 50.42 (-C-CH_3_), 51.78 (-N-CH_2_-CH_2_-O-), 63.86 (-N-CH_2_-CH_2_-O-), 65.40 (-O-CH_2_-C-N-), 65.85 (-C-CH_2_-OH-), 70.09 (C-CH_2_-O-), 125.77 (-C-CH-N-), 146.35 (-O-CH_2_-C-N-), 176.06 (-C-CO-O-) ppm. MALDI-MS: *m*/*z* = 1107.6 [M + Li^+^].

**G1**-ester-acetylne (**10**). DMAP (2.97 g, 0.0243 mol) was added to a stirred mixture of **G1** (**8**, 6.68 g, 0.0061 mol) in anhydrous DCM (267 mL) in a 500 mL round bottom flask under nitrogen. DCC (15.02 g, 0.0728 mol), pyridine (133 mL) and 4-pentynoic acid (7.14 g, 0.0728 mol) were added, and the reaction mixture was then left stirring, under nitrogen, overnight. The crude mixture was filtered and the solvent evaporated. The product was purified by column chromatography (ethyl acetate) to yield the product as a yellow oil (7.39 g, 0.0042 mol, 70% yield). ^1^H-NMR (400 MHz, CD_3_OD): δ = 1.19 (s, 3H, -C-CH_3_), 2.26 (t, 2H, -C-CH), 2.46–2.53 (m, 8H, -O-C-CH_2_-CH_2_-C-), 3.45 (s, 2H, -C-CH_2_-O-CH_2_-), 4.22 (q, 4H, -C-(CH_2_-O-C-)_2_), 4.54 (s, 2H, -C-CH_2_-O-CH_2_-C), 4.55 (t, 2H, -CH-N-CH_2_-CH_2_-), 4.73 (t, 2H, -C-CH-N-CH_2_-), 8.01 (s, 1H, -N-CH-) ppm. ^13^C{^1^H}-NMR (300 MHz, CD_3_OD): δ = 15.10 (-CH_2_-C-CH), 18.20 (-C-CH_3_), 34.31 (-CH_2_-CH_2_-C-CH), 46.57 (-C-CH_2_-O-CH_2_-C-N-), 47.70 (-C-CH_3_), 50.22 (-N-CH_2_-CH_2_-O-), 64.51 (-N-CH_2_-CH_2_-O-), 65.59 (-C-CH_2_-O-CH_2_-C-N-), 66.61 (-C-CH_2_-O-C-), 70.19 (-C-CH_2_-O-CH_2_-C-N-), 70.55 (-C-CH), 83.54 (-C-CH), 125.49 (-C-CH-N-), 146.51 (-C-CH-N-), 172.84 (-O-C-CH_2_-), 173.72 (O-C-C-) ppm. MALDI-MS: *m*/*z* = 1747.5 [M + Li^+^].

Protected Generation **2** (**pG2**, **11**). A solution of CuSO_4_·5H_2_O (0.1463 g, 0.000586 mol) in water (10 mL) was added to a round bottom flask containing a stirred solution of **10** (1.70 g, 0.0009766 mol) and **5** (2.2807 g, 0.009375 mol) in THF (10 mL). Sodium ascorbate (0.2322 g, 0.001172 mol) was added and the mixture was allowed to react overnight. The product mixture was evaporated, dissolved in a minimum of methanol and run through a silica plug to remove the copper salts. The product was then purified by dialysis (MWCO = 1000 Da, methanol) to yield a brown solid (2.05 g, 0.000556 mol, 57% yield). ^1^H-NMR (400 MHz, CD_3_OD): δ = 1.02 (s, 6H, CH_3_-C-O-CH_2_-C-CH_3_), 1.10 (s, 3H, C-C-O-CH_2_-C-CH_3_), 1.28 (s, 6H, CH_3_-C-O-CH_2_-C-CH_3_), 1.39 (s, 6H, CH_3_-C-O-CH_2_-C-CH_3_), 2.68 (t, 4H, -O-C-CH_2_-CH_2_-), 2.94 (t, 4H, t, 4H, -O-C-CH_2_-CH_2_-), 3.43 (s, 2H, -C-CH_2_-O-CH_2_-C-N-), 3.64 (dd, 4H, -C-CH_2_-O-C-CH_3_), 4.07 (dd, 4H, -C-CH_2_-O-C-CH_3_), 4.10 (s, 4H, -C-CH_2_-O-C-CH_2_), 4.50 (m, 8H, -N-CH_2_-CH_2_-O-C-C-CH_2_-O-C-CH_3_, -O-CH_2_-C-CH-N-CH_2_-CH_2_-), 4.68 (m, 6H, -N-CH_2_-CH_2_-O-C-C-CH_2_-O-C-CH_3_, -O-CH_2_-C-CH-N-CH_2_-CH_2_-), 7.85 (s, 1H, -O-CH_2_-C-CH-N-), 8.02 (s, 2H, -C-CH_2_-CH_2_-C-CH-N-) ppm. ^13^C{^1^H}-NMR (300 MHz, CD_3_OD): δ = 18.10, 18.68, 21.85, 26.49, 34.24, 43.24, 46.57, 47.67, 50.03, 50.17, 50.27, 64.15, 64.48, 65.59, 66.51, 67.04, 70.22, 99.44, 124.20, 125.51, 146.45, 147.69, 173.37, 173.68, 175.31 ppm. MALDI-MS: *m*/*z* = 3710.1[M + Na^+^].

Generation **2** (**G2**, **12**): BiCl_3_ (0.0009 g, 0.00000285 mol) and a drop of water were added to a stirred solution of **pG2** (**11**, 0.025 g, 0.00000678 mol) in acetonitrile (1.1 mL). The reaction mixture was left stirring for 60 h at 45 °C. The product mixture was evaporated, dissolved in methanol, and filtered to remove the bismuth salts. The solvent was evaporated to yield a brown oil (0.020 g, 0.00000604 mol, 89% yield). ^1^H-NMR (400 MHz, CD_3_OD): δ = 1.10 (s, 9H, -C-CH_3_), 2.69 (t, 4H, -C-CH_2_-CH_2_-C-N-), 2.95 (t, 4H, -C-CH_2_-CH_2_-C-N-), 3.43 (s, 2H, -C-CH_2_-O-CH_2_-C-N-), 3.60 (q, 8H, -C-CH_2_-OH), 4.10 (s, 4H, -C-CH_2_-O-C-), 4.48 (m, 8H, -N-CH_2_-CH_2_-O-C-C-CH_2_-OH, -O-CH_2_-C-CH-N-CH_2_-CH_2_-O-), 4.66 (m, 4H, -N-CH_2_-CH_2_-O-C-C-CH_2_-OH), 4.71 (s, 2H, -O-CH_2_-C-CH-N-CH_2_-CH_2_-O-, -O-CH_2_-C-CH-N-CH_2_-CH_2_-O-), 7.86 (s, 2H, -CH_2_-CH_2_-C-CH-N-), 8.03 (s, 1H, -O-CH_2_-C-CH-N-) ppm. ^13^C{^1^H}-NMR (300 MHz, CD_3_OD): δ = ppm 17.41, 18.08, 34.18, 46.53, 47.65, 49.99, 50.17, 50.34, 51.79, 63.91, 64.45, 65.51, 65.86, 66.51, 70.13, 124.35, 125.61, 146.37, 147.58, 173.47, 173.70, 176.08. MALDI-MS: *m*/*z* = 3388.5[M + K^+^].

**G2**-ester-acetylene (**13**). DMAP (0.1586 g, 0.001298 mol) was added to a stirred mixture of **12** (0.2732 g, 0.00008113 mol) in anhydrous DCM (6.6 mL), under nitrogen. 1-ethyl-(3-dimethylaminopropyl)-carbodiimide hydrochloride (EDC) (0.3732 g, 0.001947 mol), pyridine (3.3 mL) and 4-pentynoic acid (0.1911 g, 0.001947 mol) were added to the flask. The reaction mixture was left stirring, under nitrogen, overnight. The crude product mixture was then washed with methanol, dissolved in chloroform and filtered to yield the product as brown oil (0.27 g, 0.0000581 mol, 63% yield). ^1^H-NMR (400 MHz, CDCl_3_): δ = 1.12 (s, 3H, -N-C-CH_2_-CH_2_-C-O-CH_2_-C-CH_3_), 1.20 (s, 6H, -CH-C-CH_2_-CH_2_-C-O-CH_2_-C-CH_3_), 1.98 (s, 4H, -C-CH), 2.50 (m, 16H, -C-CH_2_-CH_2_-C-CH), 2.70 (t, 4H, -O-C-CH_2_-CH_2_-C-N-), 2.97 (t, 4H, -O-C-CH_2_-CH_2_-C-N-), 3.42 (s, 2H, -C-CH_2_-O-CH_2_-), 4.10 (m, 4H, -C-CH_2_-O-C-CH_2_-CH_2_-C-N-), 4.21 (m, 8H, -C-CH_2_-O-C-CH_2_-CH_2_-C-CH), 4.51 (m, 8H, -CH_2_-CH_2_-C-N-N-N-CH_2_-CH_2_-O-, -C-CH_2_-O-CH_2_-C-CH-N-CH_2_-CH_2_-), 4.59 (m, 4H, -C-CH_2_-O-CH_2_-C-CH-N-CH_2_-CH_2_-), 4.65 (m, 2H, -C-CH_2_-O-CH_2_-C-CH-N-CH_2_-CH_2_-), 7.48 (s, 2H, -O-C-CH_2_-CH_2_-C-CH-N-), 7.78 (s, 1H, -CH_2_-O-CH_2_-C-CH-N-) ppm. ^13^C{^1^H}-NMR (300 MHz, CDCl_3_): δ = ppm 14.26, 17.58, 17.68, 20.76, 33.11, 33.15, 43.19, 45.17, 46.27, 46.29, 48.67, 63.10, 64.75, 65.10, 65.25, 69.02, 69.34, 77.20, 82.26, 121.87, 123.31, 145.29, 146.22, 171.09, 171.98, 172.09, 172.14. MALDI-MS: *m*/*z* = 4669.1 [M + Na^+^].

**pG3** (**14**). A solution of CuSO_4_·5H_2_O (0.0081 g, 0.000586 mol) in water (2 mL) was added to a round bottom flask containing a stirred solution of **13** (0.1262 g, 0.00002715 mol) and **5** (0.1268 g, 0.0005212 mol) in THF (2 mL). Sodium ascorbate (0.0129 g, 0.00006515 mol) was added and the mixture was allowed to react overnight. The product mixture was evaporated, dissolved in a minimum of methanol and run through a silica plug to remove the copper salts. The product was precipitated out of chloroform with hexanes, and dried to yield a yellow oil (0.067 g, 0.00000785 mol, 30% yield). ^1^H-NMR (400 MHz, CDCl_3_): δ = 1.04 (s, 12H, CH_3_-C-O-CH_2_-C-CH_3_), 1.11 (s, 3H, C-C-O-CH_2_-C-CH_3_), 1.12 (s, 6H, C-C-O-CH_2_-C-CH_3_), 1.33 (s, 12H, CH_3_-C-O-CH_2_-C-CH_3_), 1.41 (s, 12H, CH_3_-C-O-CH_2_-C-CH_3_), 2.69 (t, 12H, -O-C-CH_2_-CH_2_-), 2.97 (t, 12H, -O-C-CH_2_-CH_2_-), 3.40 (s, 2H, C-CH_2_-O-CH_2_-), 3.62 (dd, 8H, -C-CH_2_-O-C-CH_3_), 4.10 (s, 12H, -C-CH_2_-O-C-CH_2_), 4.12 (dd, 8H, -C-CH_2_-O-C-CH_3_), 4.40–4.75 (m, 30H, -CH_2_-CH_2_-C-N-N-N-CH_2_-CH_2_-O-, -C-CH_2_-O-CH_2_-C-CH-N-CH_2_-CH_2_-, -N-CH_2_-CH_2_-O-C-C-CH_2_-O-C-CH_3_), 7.56 (s, 2H, -CH-N-CH_2_-CH_2_-O-C-C-CH_2_-O-C-CH_2_-), 7.61 (s, 4H, -CH-N-CH_2_-CH_2_-O-C-C-CH_2_-O-C-CH_3_), 7.88 (s, 1H, -CH_2_-O-CH_2_-C-CH-N-) ppm. ^13^C{^1^H}-NMR (300 MHz, CDCl_3_): δ = ppm 17.48, 18.12, 20.71, 20.86, 26.22, 33.05, 41.98, 46.19, 48.68, 48.99, 62.82, 62.94, 65.04, 65.85, 68.41, 77.20, 98.05, 122.22, 146.71, 171.86, 171.98, 172.05, 173.70. MALDI-MS: *m*/*z* = 8580.6 [M + K^+^].

**G3** (**15**). Dowex Cationic Resin (0.0629 g) was added to a solution of **pG3** (**14**, 0.1531 g, 0.0000179 mol) in methanol (8.5 mL). The mixture was left stirring for three days at 45 °C. The resin was then filtered off and the solvent evaporated to yield the product as a yellow oil (0.0983 g, 0.0000124 mol, 71% yield). ^1^H-NMR (400 MHz, CD_3_OD): δ = 1.09 (s, 21H, -C-CH_3_), 2.69 (m, 12H, -O-C-CH_2_-CH_2_-C-N-), 2.95 (m, 12H, -O-C-CH_2_-CH_2_-C-N-), 3.43 (s, 2H, -C-CH_2_-O-CH_2_-C-), 3.60 (q, 16H, -C-CH_2_-OH), 4.10 (m, 12H, -C-CH_2_-O-C-), 4.48 (m, 16H, -C-CH_2_-O- CH_2_-C-N-, -N-CH_2_-CH_2_-O-), 4.66 (m, 14H, -N-CH_2_-CH_2_-O-), 7.82–8.07 (m, 7H, -C-CH-N-) ppm. ^13^C{^1^H}-NMR (300 MHz, DMSO-*d*_6_): δ = ppm 16.65, 16.85, 16.89, 20.38, 32.69, 45.77, 45.80, 48.05, 48.34, 48.53, 50.24, 51.25, 62.24, 62.94, 63.06, 63.68, 63.84, 64.82, 122.31, 122.44, 123.98, 144.08, 145.22, 145.26, 171.50, 171.83, 174.20. MALDI-MS: *m*/*z* = 7894.2 [M + H^+^].

**G3**-ester-acetylene (**16**). DMAP (0.0266 g, 0.0002181 mol) was added to a stirred mixture of **G3** (0.0713 g, 0.000006814 mol) in anhydrous DCM (3.3 mL), under nitrogen. EDC (0.0627 g, 0.0003271 mol), pyridine (1.7 mL) and 4-pentynoic acid (0.0796 g, 0.0003271 mol) were added and the reaction mixture was left stirring, under nitrogen, overnight. The crude product mixture was dissolved in DCM and filtered to remove the salts. The product was then precipitated out of DCM with hexanes. Ether and hexanes washes were then performed to obtain a light brown oil (38% yield). ^1^H-NMR (400 MHz, CDCl_3_): δ = 1.07–1.24 (m, 32H, -CH_3_), 1.99 (s, 8H, -CH), 2.43–2.55 (m, 32H, -CO-CH_2_-CH_2_-C-CH), 2.71 (m, 12H, -CO-CH_2_-CH_2_-C-N-), 2.96 (m, 12H, -CO-CH_2_-CH_2_-C-N-), 3.42 (s, 2H, -C-CH_2_-O-CH_2_-C-N-), 4.11 (s, 12H, CH_3_-C-CH_2_-O-CO-CH_2_-CH_2_-C-N-), 4.17–4.26 (m, 16H, CH_3_-C-CH_2_-O-CO-CH_2_-CH_2_-C-CH), 4.49–4.66 (m, 30H, -N-CH_2_-CH_2_-O-, -C-CH_2_-O-CH_2_-C-N-), 7.48–7.80 (m, 7H, -C-CH-N-) ppm. ^13^C{^1^H}-NMR (300 MHz, CDCl_3_): δ = ppm 14.29, 17.29, 17.62, 17.72, 20.9, 29.69, 33.14, 43.60, 45.22, 46.33, 48.68, 48.98, 62.95, 63.14, 64.82 (-C-CH_2_-O-CH_2_-C-N-N-N-CH_2_-CH_2_-), 64.97, 65.14, 65.28, 65.80, 69.39, 77.21, 82.30, 121.91, 121.98, 122.38, 146.18, 146.25, 146.29, 171.12, 172.01, 172.05 172.13, 172.15, 172.18. MALDI-MS: *m*/*z* = 10,000–11,000 (theoretical mass = 10,456).

*Amine functionalization*: *tert-butyl (2-hydroxyethyl)carbamate* (**17**). Di-*tert*-butyl dicarbonate (BOC_2_O) (7.86 g, 0.036 mol) was added slowly in a portionwise fashion to a stirred solution of ethanolamine (2.0 g, 0.033 mol) in DCM and triethylamine (5 mL), at 0 °C. The mixture was allowed to react overnight, at room temperature. The crude mixture was washed with an aqueous potassium carbonate solution, followed by an aqueous hydrochloric acid solution, brine, and finally water. The organic phase was isolated, dried with magnesium sulfate, and the solvent evaporated to yield the product as a colourless oil (3.70 g, 0.023 mol, 70% yield). ^1^H-NMR (400 MHz, CDCl_3_): δ = 1.47 (s, 9H, -C-(CH_3_)_3_), 3.26 (s, 2H, -CH_2_-NH-), 3.67 (s, 2H, -CH_2_-OH), 5.04 (s, 1H, -NH-) ppm. ^13^C{^1^H}-NMR (300 MHz, CDCl_3_): δ = 28.6 (s, -C-(CH_3_)_3_), 43.4 (s, -CH_2_-NH-), 62.8 (s, -CH_2_-OH), 79.9 (C-(CH_3_)_3_), 157.1 (-NH-CO-O-) ppm.

*2-((tert-Butoxycarbonyl)amino)ethyl methanesulfonate* (**18**). Methanesulfonyl chloride (9.42 g, 0.0822 mol) was added very slowly in a dropwise fashion to a stirred solution of (**17**) (12.8713 g, 0.0798 mol) in anhydrous DCM (500 mL) and triethylamine (20 mL) at 0 °C, under nitrogen. The reaction was left stirring, under nitrogen at room temperature overnight. The crude mixture was then washed with water (2 × 250 mL) and brine (2 × 250 mL). The organic layer was isolated, dried with magnesium sulfate and the solvent evaporated to yield the product as a yellow oil (17.95 g, 0.075 mol, 94% yield). ^1^H-NMR (400 MHz, CDCl_3_): δ = 1.44 (s, 9H, -C-(CH_3_)_3_), 3.03 (s, 3H, -S-CH_3_), 3.46 (m, 2H, -CH_2_-NH-), 4.28 (m, 2H, -CH_2_-O-), 4.92 (s, 1H, -NH-) ppm. ^13^C{^1^H}-NMR (300 MHz, CDCl_3_): δ = 28.6 ( -C-(CH_3_)_3_), 37.6 (-CH_2_-NH-), 40.2 (-S-CH_3_), 69.1 (-CH_2_-O-), 80.2 (-C-(CH_3_)_3_) 158 (-NH-CO-O-) ppm.

*tert-Butyl (2-azidoethyl)carbamate* (**19**): Sodium azide (27.30 g, 0.4199 mol) was added to a stirred solution of (**18**) (19.98 g, 0.0835 mol) in DMF (100 mL) and water (100 mL), under nitrogen. The reaction was left stirring, at 80 °C overnight, under nitrogen. The crude product mixture was extracted with ethyl acetate (3 × 100 mL), the organic layers were isolated and combined and washed with water (3 × 100 mL) and brine (2 × 100 mL). The organic layer was isolated, dried with magnesium sulfate and the solvent evaporated to yield the product as a yellow oil (9.93 g, 0.0533 mol, 64% yield). ^1^H-NMR (400 MHz, CDCl_3_): δ = 1.44 (s, 9H, -C-(CH_3_)_3_), 3.28 (m, 2H, -CH_2_-NH-), 3.40 (m, 2H, N_3_-CH_2_-), 4.82 (s, 1H, -NH-) ppm. ^13^C{^1^H}-NMR (300 MHz, CDCl_3_): δ = 28.3 (-C-(CH_3_)_3_), 40.0 (-CH_2_-NH-), 51.2 (-CH_2_-N_3_), 79.8 (-C-(CH_3_)_3_), 155.70 (-NH-CO-O-) ppm.

*3-Azidopropylamine* (**20**). A solution of sodium azide (3.39 g, 0.05215 mol) in water (11 mL) was added to a stirred solution of 3-bromopropylamine hydrobromide (3.42 g, 0.01566 mol) in water (8 mL). The reaction was refluxed overnight, and 2/3 of the solvent was then evaporated and the crude mixture was placed in an ice bath. Potassium hydroxide (4.2 g, 0.0749 mol) and ether (50 mL) were added, the aqueous phase was isolated and washed with ether (3 × 35 mL). The organic phases were isolated, combined and dried with magnesium sulfate to yield the product as a yellow oil (1.38 g, 0.0138 mol, 88% yield). ^1^H-NMR (400 MHz, CDCl_3_): δ = 1.42 (s, 2H, -NH_2_), 1.72 (q, 2H, -CH_2_-CH_2_-CH_2_-), 2.79 (t, 2H, NH_2_-CH_2_-CH_2_-CH_2_-N_3_), 3.36 (t, 2H, NH_2_-CH_2_-CH_2_-CH_2_-N_3_) ppm. ^13^C{^1^H}-NMR (300 MHz, CDCl_3_): δ = 30.54 (-CH_2_-CH_2_-CH_2_-), 36.13 (N_3_-CH_2_-CH_2_-CH_2_-NH_2_), 51.12 (N_3_-CH_2_-CH_2_-CH_2_-NH_2_) ppm.

*tert-Butyl (3-azidopropyl)carbamate* (**21**). A solution of BOC_2_O (1.0899 g, 0.004994 mol) in methanol (6 mL) was added dropwise to a solution of (**20**) (0.500 g, 0.004994 mol) in triethylamine (3.48 mL) and methanol (20 mL). The reaction was left stirring, overnight at room temperature. The product mixture was filtered, and the solvent evaporated. The mixture was purified by column chromatography (chloroform) to yield the product as a yellow oil (0.5457 g, 0.00273 mol, 55% yield). ^1^H-NMR (400 MHz, CDCl_3_): δ = 1.41(s, 9H, -C-(CH_3_)_3_), 1.74 (q, 2H, -CH_2_-CH_2_-CH_2_-), 3.17 (t, 2H, -CH_2_-CH_2_-CH_2_-N_3_), 3.33 (t, 2H, -CH_2_-CH_2_-CH_2_-N_3_), 4.75 (s, 1H, -NH-) ppm. ^13^C{^1^H}-NMR (300 MHz, CDCl_3_): δ = 28.39 (C-(CH_3_)_3_), 29.87 (-CH_2_-CH_2_-CH_2_-), 48.13 (N_3_-CH_2_-CH_2_-CH_2_-NH-), 53.93 (N_3_-CH_2_-CH_2_-CH_2_-NH-), 79.74 (-C-(CH_3_)_3_), 155.47 (-NH-COO-) ppm.

**G0**-NHBOC. A solution of CuSO_4_·5H_2_O (0.0754 g, 0.000302 mol) in water (2.5 mL) was added to a round bottom flask containing a stirred solution of **7** (0.300 g, 0.00104 mol) and **19** (0.8528 g, 0.00458 mol) in THF (2.5 mL). Sodium ascorbate (0.1236 g, 0.000624 mol) was added and the mixture was allowed to react overnight. The product mixture was evaporated and purified by column chromatography (acetone) to yield an orange solid (0.9041 g, 0.000875 mol, 97% yield). ^1^H-NMR (400 MHz, CDCl_3_): δ = 1.36 (s, 9H, -C-(CH_3_)_3_), 3.41 (s, 2H, -C-CH_2_-O-), 3.55 (q, 2H, -N-CH_2_-CH_2_-NH-), 4.43 (t, 2H, -N-CH_2_-CH_2_-NH-), 4.47 (s, 2H, -C-CH_2_-O-CH_2_-), 5.62 (t, 1H, -NH-), 7.56 (s, 1H,-C-CH-N- ) ppm. ^13^C{^1^H}-NMR (300 MHz, CDCl_3_): δ = 28.66 (-C-(CH_3_)_3_), 40.29 (-CH-N-CH_2_-CH_2_-), 46.47 (-C-CH_2_-O-CH_2_-C-N-), 48.52 (-CH-N-CH_2_-CH_2_-), 65.44 (-C-CH_2_-O-CH_2_-C-N-), 70.25 (-C-CH_2_-O-CH_2_-C-N-), 80.21 (-C-(CH_3_)_3_), 126.40 (-CH-), 147.20 (-C-CH-), 154.93 (-CO-) ppm. ESI-MS: *m*/*z* = 1056.5 [M + Na^+^].

**G1**-NHBOC. A solution of CuSO_4_·5H_2_O (0.0081 g, 0.00003232 mol) in water (2 mL) was added to a round bottom flask containing a stirred solution of **10** (0.0987 g, 0.0000567 mol) and **20** (0.1655 g, 0.0006804 mol) in THF (2 mL). Sodium ascorbate (0.0135 g, 0.00006804 mol) was added and the mixture was allowed to react overnight. The product mixture was evaporated and purified by column chromatography (acetone) to yield a sticky yellow solid (0.095 g, 0.0000284 mol, 50% yield). ^1^H-NMR (400 MHz, CD_3_OD): δ = 1.09 (s, 3H, -C-C-CH_3_), 1.42 (s, 18H, -C-(CH_3_)_3_), 2.03 (m, 4H, -N-CH_2_-CH_2_-CH_2_-NH-), 2.68 (t, 4H, -O-C-CH_2_-CH_2_-C-N-), 2.94 (t, 4H, -O-C-CH_2_-CH_2_-C-N-), 3.05 (t, 4H, -N-CH_2_-CH_2_-CH_2_-NH-), 3.42 (s, 2H, -C-CH_2_-O-), 4.09 (q, 4H, CH_3_-C-CH_2_-O-), 4.38 (t, 4H, -N-CH_2_-CH_2_-CH_2_-NH-), 4.51 (m, 4H, -CH_2_-O-CH_2_-C-CH-N-CH_2_-), 4.71 (t, 2H, -N-CH_2_-CH_2_-O-), 6.69 (t, 2H, -NH-), 7.77 (s, 2H, -CH-N-CH_2_-CH_2_-CH_2_-), 8.03 (s, 1H, -CH_2_-O-CH_2_-C-CH-N-) ppm. ^13^C{^1^H}-NMR (300 MHz, CDCl_3_): δ = 18.08 (CH_3_-C-CH2-), 21.84 (-CH_2_-CH_2_-C-N-), 28.87 (-C-(CH_3_)_3_), 31.67 (-CH_2_-CH_2_-NH-), 34.22 (-CH_2_-CH_2_-C-N-), 38.52 (-CH_2_-NH-), 46.55 (-C-CH_2_-O-CH_2_-C-N), 47.67 (-CH_2_-C-CH_3_), 49.90 (-N-CH_2_-CH_2_-O-), 50.16 (-N-CH_2_-CH_2_-CH_2_-NH-), 64.45 (-N-CH_2_-CH_2_-O-), 65.58 (-C-CH_2_-O-CH_2_-C-N), 66.48 (-CH_2_-C-CH_3_), 70.18 (-C-CH_2_-O-CH_2_-C-N), 80.12 (-C-(CH_3_)_3_), 123.73 (-C-CH-N-CH_2_-CH_2_-CH_2_-), 125.52 (-C-CH_2_-O-CH_2_-C-CH-N-), 146.45 (-C-CH_2_-O-CH_2_-C-CH-N-), 147.43 (-C-CH-N-CH_2_-CH_2_-CH_2_-), 158.45 (-CO-O-C-(CH_3_)_3_), 173.43 (-O-CO-CH_2_-), 173.67 (-O-CO-C-) ppm. MALDI-MS: *m*/*z* = 3365.1 [M + Na^+^].

**G2**-**NHBOC**. A solution of CuSO_4_·5H_2_O (0.0059 g, 0.00002349 mol) in water (1 mL) was added to a round bottom flask containing a stirred solution of **13** (0.091 g, 0.00001958 mol) and **20** (0.0941 g, 0.0004698 mol) in THF (1 mL). Sodium ascorbate (0.0093 g, 0.00004698 mol) was added and the mixture was allowed to react overnight. The product mixture was evaporated and filtered in DCM to remove the salts. The product was precipitated out of DCM using ether, isolated and dried to yield a viscous yellow oil (0.0429 g, 0.000005463 mol, 28% yield). ^1^H-NMR (400 MHz, CDCl_3_): δ = 1.11 (s, 9H, -O-C-C-CH_3_), 1.41 (s, 36H, -C-(CH_3_)_3_), 2.04 (q, 8H, -N-CH_2_-CH_2_-CH_2_-NH-), 2.69 (t, 12H, -O-C-CH_2_-CH_2_-C-N-), 2.97 (t, 12H, -O-C-CH_2_-CH_2_-C-N-), 3.10 (m, 8H, -N-CH_2_-CH_2_-CH_2_-NH-), 3.46 (s, 2H, -C-CH_2_-O-CH_2_-), 4.10 (m, 12H, CH_3_-C-CH_2_-O-C-), 4.36 (t, 8H, -N-CH_2_-CH_2_-CH_2_-NH-), 4.48 (m, 8H, -CH_2_-CH_2_-C-N-N-N-CH_2_-CH_2_-O-), 4.58 (t, 4H, -CH_2_-O-CH_2_-C-N-N-N-CH_2_-CH_2_-O-), 4.65 (s, 2H, -C-CH_2_-O-CH_2_-), 5.14 (s, 4H, -NH-), 7.47 (s, 4H, -CH-N-CH_2_-CH_2_-CH_2_-NH-), 7.54 (s, 2H, -C-CH_2_-CH_2_-C-CH-N-CH_2_-CH_2_-O-), 7.83 (s, 1H, -O-CH_2_-C-CH-N-) ppm. ^13^C{^1^H} NMR (300 MHz, CDCl_3_): δ = 17.58 (-CH_2_-C-CH_3_), 20.76 (-CO-CH_2_-CH_2_-C-CH-N-CH_2_-CH_2_-O-), 20.81 (-CH_2_-C-CH-N-CH_2_-CH_2_-CH_2_-), 28.36 (-C-(CH_3_)_3_), 30.62 (-N-CH_2_-CH_2_-CH_2_-), 33.17 (-CO-CH_2_-CH_2_-C-CH-N-CH_2_-CH_2_-O-), 33.28 (-CH_2_-CH_2_-C-CH-N-CH_2_-CH_2_-CH_2_-), 37.36 (-N-CH_2_-CH_2_-CH_2_-), 45.18 (-C-CH_2_-O-CH_2_-C-N-), 46.26 (-C-CH_2_-O-CH_2_-C-CH-N-CH_2_-CH_2_-), 46.30 (-CO-CH_2_-CH_2_-C-CH-N-CH_2_-CH_2_-O-), 47.47 (-N-CH_2_-CH_2_-CH_2_-), 48.68 (-C-CH_2_-O-CO-CH_2_-CH_2_-C-CH-N-CH_2_-CH_2_-CH_2_-), 48.75 (-C-CH_2_-O-CO-CH_2_-CH_2_-C-CH-N-CH_2_-CH_2_-O-), 63.05 (-CO-CH_2_-CH_2_-C-CH-N-CH_2_-CH_2_-O-), 63.11 (-C-CH_2_-O-CH_2_-C-CH-N-CH_2_-CH_2_-), 64.75 (-C-CH_2_-O-CH_2_-C-N-), 65.14 (-C-CH_2_-O-CO-CH_2_-CH_2_-C-CH-N-CH_2_-CH_2_-CH_2_-), 69.05 (-C-CH_2_-O-CH_2_-C-N-), 70.50 (-C-CH_2_-O-CO-CH_2_-CH_2_-C-CH-N-CH_2_-CH_2_-O-), 79.32 (-C-(CH_3_)_3_), 121.67 (-C-CH-N-CH_2_-CH_2_-CH_2_-), 122.10 (-CO-CH_2_-CH_2_-C-CH-N-CH_2_-CH_2_-O-), 123.50 (-C-CH_2_-O-CH_2_-C-CH-N-), 146.01 (-CO-CH_2_-CH_2_-C-CH-N-CH_2_-CH_2_-O-), 146.15 (-C-CH-N-CH_2_-CH_2_-CH_2_-), 149.93 (-C-CH_2_-O-CH_2_-C-CH-N-), 156.12 (-CO-O-C-(CH_3_)_3_), 171.96 (-CO-CH_2_-CH_2_-C-CH-N-CH_2_-CH_2_-O-), 172.01 (-CO-CH_2_-CH_2_-C-CH-N-CH_2_-CH_2_-CH_2_-), 172.12 (-CO-CH_2_-CH_2_-C-CH-N-CH_2_-CH_2_-O-CO-), 172.15 (-C-CH_2_-O-CH_2_-C-CH-N-CH_2_-CH_2_-O-CO-) ppm. MALDI-MS: *m*/*z* = 7881.9 [M + Na^+^].

**G0**-NH_3_^+^ (**22**). TFA (5 mL) was added dropwise to a solution of **G0-NHBOC** (0.9041 g, 0.000875 mol) in DCM (5 mL). The reaction was left stirring for 5 min, the solvent was evaporated and the product was dissolved in methanol. The methanol was evaporated, bringing the remaining TFA with it. This methanol addition and evaporation cycle was then repeated several times to ensure that all remaining TFA had been evaporated. The product was obtained as a sticky orange solid (0.800 g, 0.000735 mol, 84% yield). ^1^H-NMR (400 MHz, CD_3_OD): δ = 1.80 (s, 3H, -NH_3_), 3.47 (s, 2H, -C-CH_2_-O-), 3.59 (t, 2H, -N-CH_2_-CH_2_-NH_3_), 4.53 (s, 2H, -C-CH_2_-O-CH_2_-), 4.76 (t, 2H, -N-CH_2_-CH_2_-NH_3_), 8.06 (s, 1H, -C-CH-N-) ppm. ^13^C{^1^H}-NMR (300 MHz, CD_3_OD): δ = 40.30 (-CH-N-CH_2_-CH_2_-), 46.48 (-C-CH_2_-O-CH_2_-C-N-), 48.54 (-CH-N-CH_2_-CH_2_-), 65.46 (-C-CH_2_-O-CH_2_-C-N-), 70.27 (-C-CH_2_-O-CH_2_-C-N-), 119.20 (-CF_3_), 126.40 (-CH-), 147.22 (-C-CH-), 162.95 (-CO-) ppm. ESI-MS: *m*/*z* = 633.3 [M − 4TFA + H^+^].

**G1**-NH_3_^+^ (**23**). TFA (0.5 mL) was added dropwise to a solution of **G1-NHBOC** (0.05 g, 0.000015 mol) in DCM (0.5 mL). The reaction was left stirring for 5 min, the solvent was evaporated and the product was dissolved in methanol. The methanol was then evaporated, bringing the remaining TFA with it. This methanol addition and evaporation cycle was then repeated several times to ensure that all remaining TFA had been evaporated. The product was obtained as a sticky yellow solid (0.041 g, 0.000012 mol, 80% yield). ^1^H-NMR (400 MHz, CD_3_OD): δ = 1.10 (s, 3H, -C-CH_3_), 2.27 (q, 4H, -N-CH_2_-CH_2_-CH_2_-NH_3_), 2.69 (t, 4H, -O-C-CH_2_-CH_2_-C-N-), 2.95 (t, 4H, -O-C-CH_2_-CH_2_-C-N-), 3.00 (t, 4H, -N-CH_2_-CH_2_-CH_2_-NH_3_), 3.42 (s, 2H, -C-CH_2_-O-CH_2_-), 4.08 (s, 4H, CH_3_-C-CH_2_-O-), 4.51 (t, 8H, -CH_2_-O-CH_2_-C-), 4.71 (t, 2H,-N-CH_2_-CH_2_-O-C-, -N-CH_2_-CH_2_-CH_2_-NH_3_), 7.80 (s, 2H, -CH-N-CH_2_-CH_2_-CH_2_-), 8.04 (s, 1H,-CH_2_-O-CH_2_-C-CH-N-) ppm. ^13^C{^1^H}-NMR (300 MHz, CD_3_OD): δ = 18.03 (CH_3_-C-CH2-), 21.74 (-CH_2_-CH_2_-C-N-), 29.18 (-CH_2_-CH_2_-NH_3_), 34.14 (-CH_2_-CH_2_-C-N-), 38.17 (-CH_2_-NH_3_), 46.55 (-C-CH_2_-O-CH_2_-C-N-), 47.63 (-CH_2_-C-CH_3_), 49.90 (-N-CH_2_-CH_2_-O-), 50.20 (-N-CH_2_-CH_2_-CH_2_-NH_3_-), 64.44 (-N-CH_2_-CH_2_-O-), 65.48 (-C-CH_2_-O-CH_2_-C-N-), 66.41 (-CH_2_-C-CH_3_), 70.15 (-C-CH_2_-O-CH_2_-C-N), 119.08 (-C-F_3_), 123.91 (-C-CH-N-CH_2_-CH_2_-CH_2_-), 125.64 (-C-CH_2_-O-CH_2_-C-CH-N-), 146.39 (-C-CH_2_-O-CH_2_-C-CH-N-), 147.69 (-C-CH-N-CH_2_-CH_2_-CH_2_-), 162.57 (-CO-O-C-F_3_), 173.46 (-O-CO-CH_2_-), 173.69 (-O-CO-C-) ppm. MALDI-MS: *m*/*z* = 2563.6 [M − 8TFA + Na^+^].

**G2**-NH_3_^+^ (**24**). TFA (1 mL) was added dropwise to a solution of **G2-NHBOC** (0.0429 g, 0.000005463 mol) in DCM (1 mL). The reaction was left stirring for 5 min, the solvent was evaporated and the product was dissolved in methanol. The methanol was then evaporated, bringing the remaining TFA with it. This methanol addition and evaporation cycle was repeated several times to ensure that all remaining TFA had been evaporated. The product was obtained as a sticky yellow solid (0.034 g, 0.00000421 mol, 77% yield). ^1^H-NMR (400 MHz, CD_3_OD): δ = 1.10 (s, 9H, -O-C-C-CH_3_), 2.27 (q, 8H, -N-CH_2_-CH_2_-CH_2_-NH_3_), 2.68 (t, 12H, -O-C-CH_2_-CH_2_-C-N-), 2.97 (t, 12H, -O-C-CH_2_-CH_2_-C-N-), 3.01 (m, 8H, -N-CH_2_-CH_2_-CH_2_-NH_3_), 3.41 (s, 2H, -C-CH_2_-O-CH_2_-), 4.08 (m, 12H, CH_3_-C-CH_2_-O-C-), 4.50 (m, 8H, -CH_2_-CH_2_-C-N-N-N-CH_2_-CH_2_-O-, -N-CH_2_-CH_2_-CH_2_-NH_3_), 4.66 (t, 4H, -CH_2_-O-CH_2_-C-N-N-N-CH_2_-CH_2_-O-), 4.70 (s, 2H, -C-CH_2_-O-CH_2_-), 4.88 (s, 12H, -NH_3_), 7.82 (s, 4H, -CH-N-CH_2_-CH_2_-CH_2_-NH-), 7.86 (s, 2H, -C-CH_2_-CH_2_-C-CH-N-CH_2_-CH_2_-O-), 8.07 (s, 1H, -O-CH_2_-C-CH-N-)ppm. ^13^C{^1^H}-NMR (300 MHz, CD_3_OD): δ = 16.55 (-CH_2_-CH_2_-CH_2_-N-N-N-C-CH_2_-CH_2_-CO-O-CH_2_-C-CH_3_), 16.63 (O-CH_2_-CH_2_-N-N-N-C-CH_2_-CH_2_-CO-O-CH_2_-C-CH_3_), 20.30 (-CH_2_-C-CH-N-CH_2_-CH_2_-CH_2_-), 20.35 (-CO-CH_2_-CH_2_-C-CH-N-CH_2_-CH_2_-O-), 27.71 (-N-CH_2_-CH_2_-CH_2_-), 32.70 (-CO-CH_2_-CH_2_-C-N-), 36.72 (-N-CH_2_-CH_2_-CH_2_-), 45.05 (-C-CH_2_-O-CH_2_-C-N-), 46.17 (-CO-CH_2_-CH_2_-C-CH-N-CH_2_-CH_2_-O-), 46.19 (-C-CH_2_-O-CH_2_-C-CH-N-CH_2_-CH_2_-), 46.85 (-N-CH_2_-CH_2_-CH_2_-), 48.73 (CH_3_-C-CH_2_-O-CO-), 62.97 (-N-CH_2_-CH_2_-O-), 64.05 (-C-CH_2_-O-CH_2_-C-N-), 64.97 (-C-CH_2_-O-CO-CH_2_-CH_2_-C-CH-N-CH_2_-CH_2_-CH_2_-), 65.05 (-C-CH_2_-O-CO-CH_2_-CH_2_-C-CH-N-CH_2_-CH_2_-O-), 68.75 (-C-CH_2_-O-CH_2_-C-N-), 118.50 (-CF_3_), 122.48 (-C-CH-N-), 146.08 (-C-CH-N-), 161.39 (-CO-O-CF_3_), 171.98 (-CO-O-CH_2_-C-CH_3_), 172.24 (-CO-CH_2_-CH_2_-C-CH-N-CH_2_-CH_2_-O-CO-), 172.29 (-C-CH_2_-O-CH_2_-C-CH-N-CH_2_-CH_2_-O-CO-) ppm.

### 3.3. Determination of MIC

Aqueous solutions with dendrimer concentrations ranging from 0.025–64 mg/L were prepared. Initial testing was performed with these solutions, with subsequent tests carried out with a more narrow range around the initially determined MIC, in order to increase accuracy. Mueller Hinton Broth (MHB) (85 mL) was inoculated with one colony of *Escherichia coli* (*E. coli*) ATCC11229 and incubated with shaking (150 rpm) at 37 °C, for 18–24 h. Fresh MHB (85 mL) was then inoculated with overnight culture (300 µL) and incubated for 2 h, with shaking (150 rpm), at 37 °C. The OD of the culture was then measured at 625 nm. The bacterial solution was subsequently diluted in water until its OD lay in the range of 0.08–0.13, and then 100-fold in 15% MHB/85% H_2_O. This diluted solution of bacteria (3 mL) was then added in a 1:1 ratio to the prepared dendrimer solutions, in duplicate. The initial OD at 625 nm of each dendrimer treated bacteria sample was then measured. Subsequently, the samples were incubated with shaking (150 rpm) at 37 °C, for 18–24 h, when the OD was again measured. Bacterial controls ensured that each sample contained 5 × 10^5^ colony forming units (CFU). Experiments were repeated to ensure reproducibility. The OD values of the bacterial solutions taken 18–24 h after dendrimer addition relative to the initial OD were plotted as a function of dendrimer concentration. The MIC was evaluated as the concentration where the relative OD drops sharply to zero.

### 3.4. Determination of MBC

The same dendrimer treated bacterial samples used to evaluate the MIC were also employed to determine the MBC. After their OD had been measured at 625 nm, 18–24 h after exposure, the samples were plated on agar. The MBC was determined as the lowest concentration showing no bacterial growth after incubation for 18–24 h at 37 °C.

## 4. Conclusions

We have developed a synthetic methodology to hydroxide and acetylene terminated dendrimers that can be easily functionalized with a variety of surface groups including cationic amine moieties for application as bactericides. The introduction of tetravalent core molecule in the design of dendrimers reduces the number of generations required to obtain the appropriate number of functional groups at their periphery. It also reduces the number of steps required in their synthesis, and it may facilitate their large scale preparation. Availability of terminal acetylene groups allows covalent linking of any desired functional group using “click” chemistry. We have demonstrated the introduction of cationic end groups, and have evaluated the potential of these dendrimers as bactericides. However, the scope of functionalization is not limited, and these dendrimers offer an advantageous platform to design nanocarriers for varied applications in a diverse range of areas. Our studies suggest that while designing antimicrobial dendrimers, the dendrimer generation, the functional groups at the periphery and their permeability (and thus hydrophilicty) plays an important role in enhancing their efficacy.

## Figures and Tables

**Table 1:** MIC and MBC values of dendrimers for *E. coli* (ATCC 11229^TM^ strain).

Structure	Type of End Groups	No. of End Groups	M_W_ (g/mol)	MIC (mg/L)	MIC (µM)	MBC (mg/L)	MBC (µM)
G1-OH	-OH	8	1101.1	>64	>58	>64	>58
G0-NH_3_^+^	-NH_3_^+^ TFA^−^	4	1088.5	32–64	29–59	>64	>59
G1-NH_3_^+^	-NH_3_^+^ TFA^−^	8	3454.9	0.9 ± 0.1	0.26 ± 0.03	4–8	1.1–2.3
G2-NH_3_^+^	-NH_3_^+^ TFA^−^	16	8075.0	1–16	0.12–2	-	-

**Table 2:** Comparative MIC values for dendrimers.

Generation & Backbone	No. End Groups	Type of End Groups	MW (g/mol)	MIC (mg/L)	MIC (µM)	Type of Bacteria
G3 PAMAM	31	-NH_2_	6859	6.3	0.92	*Pseudomonas aeruginosa* (PA)
G5 PAMAM	110	-NH_2_	26 373	12.5	0.47	PA
G1 Carbosilane	4	-N(Me)_3_^+^ I^−^	1470.4	4	2.7	*Escherichia coli* (*E coli*)
G2 Carbosilane	8	-N(Me)_3_^+^ I^−^	3493.8	64	18.3	*E coli*
